# Successful Pregnancy Outcome after Open Strassman Metroplasty for Bicornuate Uterus

**DOI:** 10.1155/2018/4579736

**Published:** 2018-05-31

**Authors:** Edgar Gulavi, Steve Kyende Mutiso, Charles Mariara Muriuki, Abraham Mukaindo Mwaniki

**Affiliations:** ^1^Obstetrics and Gynecology, Aga Khan University Hospital, Nairobi, Kenya; ^2^Kijabe Mission Hospital, Kiambu, Kenya; ^3^Aga Khan University Hospital, Nairobi, Kenya

## Abstract

**Introduction:**

Müllerian duct anomalies represent a group of congenital malformations that result from failure to complete bilateral paramesonephric duct elongation, fusion, canalization, or septal resorption. These anomalies are rare in the general population with a bicornuate or didelphys uterus being among the common ones. Bicornuate uterine malformations are of clinical significance due to their adverse reproductive outcomes. Metroplasty has been shown to improve reproductive outcomes of bicornuate uterine malformations. We document a case of bicornuate uterus that was managed with Strassman metroplasty and a subsequent successful pregnancy outcome.

**Case:**

A Black African lady was seen with a history of six prior miscarriages. Her diagnostic workup revealed a bicornuate uterus for which she had a Strassman metroplasty performed. She later conceived and was followed up to term with a successful live birth.

**Conclusion:**

Strassman metroplasty is a rare procedure in Sub-Saharan Africa and this case seeks to add to the body of knowledge on surgical management of Müllerian duct anomalies specifically bicornuate uterus in this region. This case report aims to increase the awareness of Müllerian duct abnormalities specifically bicornuate uterus in cases of recurrent miscarriages and highlight the diagnostic strategies to investigate and to demonstrate management options in low resource settings.

## 1. Introduction

Müllerian duct anomalies represent a group of congenital malformations that result from failure to complete bilateral paramesonephric duct elongation, fusion, canalization, or septal resorption [1]. These anomalies can be classified according to the American Society of Reproductive Medicine (ASRM) as segmental Müllerian hypoplasia or agenesis (Group I), unicornuate uterus (Group II), uterine didelphys (Group III), bicornuate uterus (Group IV), septate uterus (Group V), arcuate uterus (Group VI), and diethylstilbestrol related anomalies (Group VII) [2].

The prevalence of these malformations in general population is approximately 5%. It is 5–10% in those suffering from recurrent miscarriages and greater than one in four in women with late pregnancy losses and preterm deliveries [3]. Among the Müllerian duct anomalies, bicornuate and didelphic uteri are the more common forms, with a prevalence of 25 and 11%, respectively. Moreover, they are associated with recurrent first and second trimester miscarriages as well as higher rates of preterm delivery [3].

Bicornuate uterus is caused by incomplete fusion of the Müllerian ducts. It is characterized by two separate but communicating endometrial cavities and a single uterine cervix. Failed fusion may extend to the cervix resulting in a complete bicornuate uterus or may be partial, causing a milder abnormality. Pregnancy outcomes in patients with these anomalies are not favorable. Previous reports have demonstrated that those with bicornuate uterus have higher rates of miscarriages and preterm delivery [36% and 23%, respectively] [3–5]. The clinical presentation of bicornuate uterus includes menstrual dysfunction, primary infertility, recurrent miscarriages, preterm deliveries, and late pregnancy losses. A multidisciplinary approach is recommended in the management of patients diagnosed with Müllerian duct anomalies with input from a psychologist, an endocrinologist, and consultant gynecologist adept at such cases. With that in mind, surgical intervention remains the standard of care for those with Müllerian duct anomalies suffering from recurrent pregnancy loss or poor pregnancy outcomes [4]. Strassman metroplasty is the standard surgical procedure for correction of bicornuate uteri [5]. Paul Strassman in 1907 reported the first surgical correction for the double uterus by performing an anterior colpotomy in a patient with 8 pregnancy losses [5].

Uterine malformations are rare occurrences worldwide and surgical management of these cases is not well documented in Sub-Saharan Africa perhaps due to challenges in diagnosis or treatment. We present a case of a patient with a bicornuate uterus that was surgically managed with open Strassman metroplasty with a successful subsequent pregnancy.

## 2. Case

### 2.1. Patient Information

A 35-year-old para 0+6 Black African lady presented with a history of five first and one second trimester recurrent pregnancy losses. In addition, she had a nine-year history of irregular heavy bleeding associated with dysmenorrhea.

Her menarche was at 17 years of age with regular painful cycles that lasted 10 days. She was not on any contraceptive method and did not report any dyspareunia or urinary symptoms. Her first miscarriage occurred 11 years and was surgically managed by dilatation and curettage. Subsequently she noted changes in her menstrual cycle. Her menstrual cycle became irregular with a heavy flow for 10 days associated with severe dysmenorrhea and bowel symptoms of bloating and diarrhea. She used tranexamic acid one gram three times a day during her menses for the heavy prolonged periods and Mefenamic Acid 500 milligrams three times a day for the dysmenorrhea with reported relief of the symptoms.

### 2.2. Clinical Findings

The physical examination was unremarkable except for mild tenderness in the suprapubic region; she had grossly normal external genitalia and normal looking cervix.

### 2.3. Diagnostic Assessment

Her initial hormonal profile was as follows:

  FSH: 4.5 IU/m (3.1-7.9 IU/L)
  LH: 10 IU/L (1-18 IU/L)

She had a recent pap smear one year ago that was normal.

**Figure 1:**
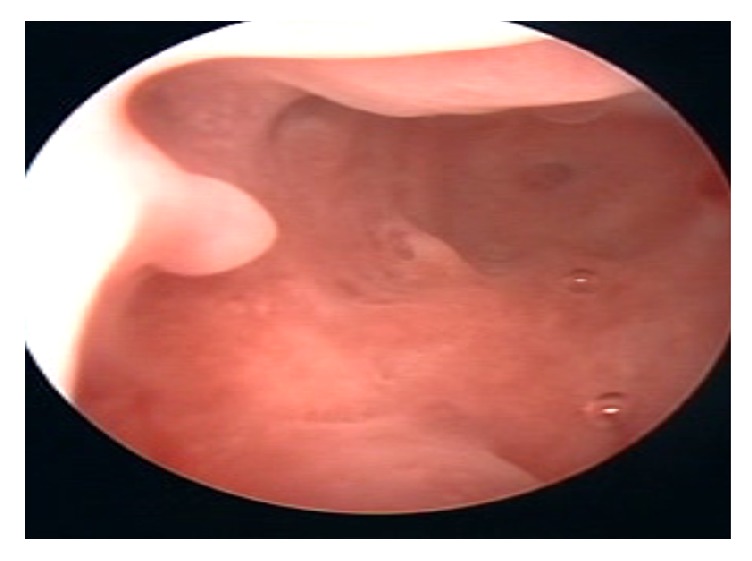
Hysteroscopy findings.

Transabdominal and transvaginal (TVS) scans had shown separate right and left cornu with multiple cysts in the peripheral ovarian parenchyma features suggestive of bicornuate uterus and polycystic ovaries (Figure 1). She also had a hysterosalpingogram (HSG) that was reported to have a uterus opacified with banana configuration oriented to the right with no delineation of fallopian tubes, findings suggestive of a unicornuate uterus.

Her past medical history was not significant. She was recently divorced, a condition she attributed to her history of several miscarriages in the context of an African cultural expectation of siring children.

She was subsequently scheduled her for hysteroscopy and diagnostic laparoscopy. She also gave consent for possible open Strassmans metroplasty.

### 2.4. Therapeutic Intervention

**Figure 2:**
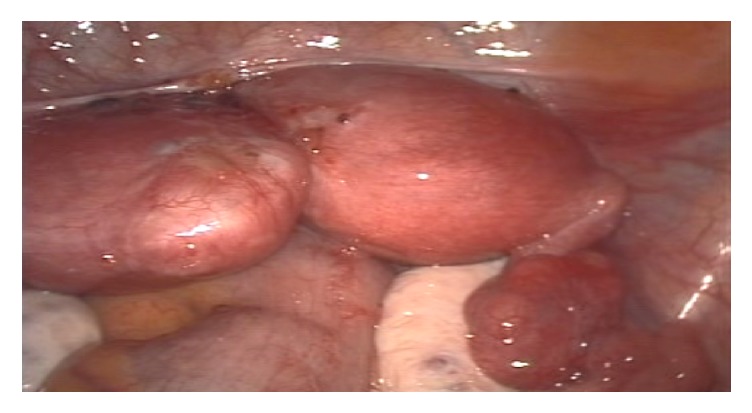
Laparoscopy findings.

**Figure 3:**
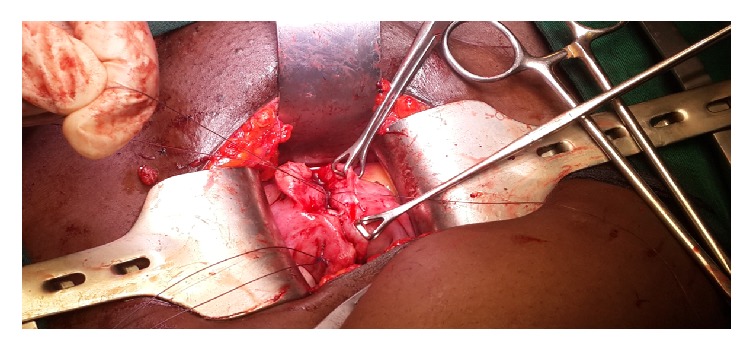
Ongoing metroplasty.

**Figure 4:**
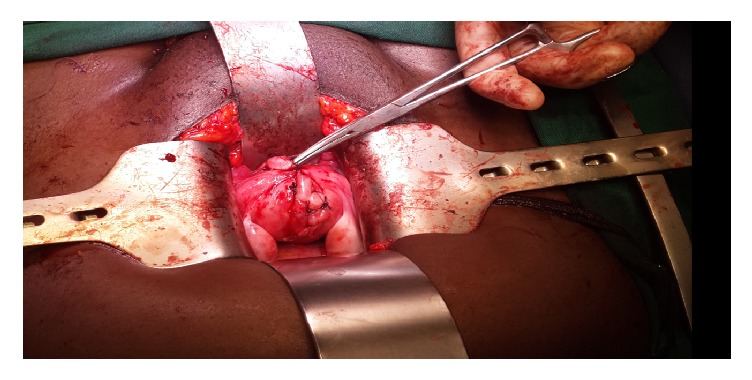
After metroplasty.

On hysteroscopy, a normal looking endometrium was visualized with a right sided tubal ostia and a small tubal ostia on the left that did not appear patent (Figure 1). Laparoscopically, a bicornuate uterus was found (Figure 2) with associated endometriosis at the vesicouterine fold. Open metroplasty was done (Figures 3 and 4). A fundal transverse incision was made and dissection done to the level of the endometrium after injection of subserosal vasopressin. Apposition of the two horns was done and the uterus sutured in layers (Figures 3 and 4). A copper intrauterine device was left in situ to separate the uterine walls and possibly reduce the chances of uterine synechiae though the evidence for this practice is still not conclusive. In addition, endometriotic deposits discovered at the vesico uterine fold were ablated.

### 2.5. Follow-Up and Outcomes

She had an unremarkable follow-up after surgery with no complications reported.

A HSG 6 months after surgery revealed unification of the uterine horns but both tubes were not delineated.

Patient was lost to follow-up but two years later she presented having conceived spontaneously and a TVS revealed a single intrauterine pregnancy at 13 weeks. Her antenatal workup was as highlighted in Table 1.

She was subsequently followed up at the antenatal clinic and had no concerns till 33 weeks when she presented with a history of lower abdominal pain worsening over the past few weeks and right sided fundal tenderness pain score of seven out of ten on the pain assessment scale. The main concern at the time of assessment was possible uterine rupture. She did not have any features suggestive of bowel obstruction as she was passing stool and bowel sounds were present. She had a normal NST and an obstetric ultrasound that was done showed a single intrauterine pregnancy with normal biophysical profile and no features of placental abruption or slower uterine segment site dehiscence. She had no other investigations done at this time. The pain was significant enough to warrant an admission for which she subsequently received antenatal steroids for lung maturity and opioid analgesia for pain relief. However, the pain did not completely resolve and she underwent a caesarean delivery at 33 weeks and 5 days due to persistent lower abdominal pain. The outcome was a live male infant with a birth weight of 1950 grams and APGAR of 9, 10, 10. There was no evidence of uterine rupture although she had venous congestion and massive varicose veins bilaterally at the cornual areas. The previous metroplasty scar was intact.

She had a resolution of the abdominal pain postoperatively and had an unremarkable postoperative recovery. She was discharged on the fifth postoperative day.

## 3. Discussion

Müllerian duct anomalies represent a group of congenital malformations that result from failure to complete bilateral duct elongation, fusion, canalization, or septal resorption [1].

Various classification schemes for female reproductive tract anomalies exist, but the most common classification was proposed by Buttram and Gibbons (1979) and adapted by the American Society for Reproductive Medicine (ASRM) (former American Fertility Society, 1988). Within this system, six groups are elucidated: segmental Müllerian hypoplasia or agenesis (Group I), unicornuate uterus (Group II), uterine didelphys (Group III), bicornuate uterus (Group IV), septate uterus (Group V), arcuate uterus (Group VI), and diethylstilbestrol related anomalies (Group VII) [2]. The case highlighted above of bicornuate uterus fell into Group IV ASRM classification.

Among the Müllerian duct anomalies, bicornuate and didelphic uteri are common forms, with the prevalence of 25 and 11%.

There are no specific risk factors highlighted in literature that predispose to these abnormalities. Uterine congenital anomalies have a heterogeneous genetic basis, with implications of Wilms tumour 1 gene* (WT1)*, paired box gene 2* (Pax2), WNT2*, pre-B-cell leukemia transcription factor 1* (PBX1)*, and homeobox* (HOX)* genes [6]. In the case highlighted above no risk factor was found.

Most cases are diagnosed during evaluation for obstetric or gynecologic conditions, but in the absence of symptoms, most anomalies remain undiagnosed [6]. In this particular case, a history of first and second trimester miscarriages as well as menstrual irregularities warranted clinical evaluation and investigation.

The question of whether Müllerian anomalies are significantly more often combined with endometriosis is a controversially discussed problem. Some publications described this association in patients with obstructive but not nonobstructive Müllerian anomalies or controls without Müllerian anomalies. It is well known that obstructive Müllerian anomalies are significantly more often associated with endometriosis, a disease with an adverse effect on fertility. The underlying pathophysiological mechanism could be the increased risk of retrograde menstruation [7, 8]. Radiologic discrimination of bicornuate uterus from the septate uterus and unicornuate uterus with a horn can be challenging as was in the case highlighted here where initial HSG was suggestive of a unicornuate uterus. However, it is important to distinguish them because septate uterus is easily treated with hysteroscopic septal resection. Widely diverging horns seen on HSG may suggest a bicornuate uterus. An intercornual angle greater than 105 degrees suggests bicornuate uterus, whereas one less than 75 degrees indicates a septate uterus [9]. However, MRI may be necessary to define fundal contour. With this, an intrafundal downward cleft measuring greater than or equal to 1 cm is indicative of bicornuate uterus, whereas a cleft depth < 1 cm indicates a septate uterus [9]. Use of 3D sonography also allows internal and external uterine assessment. Thus, sonography and HSG seem acceptable imaging techniques in the initial investigation. When the presumptive diagnosis is a septate uterus, laparoscopy may be performed for a definitive diagnosis and before hysteroscopic resection of the septum is initiated if in doubt. In this present case, HSG and TVS combined with findings from a hysteroscopy and laparoscopy were sufficient to make the diagnosis.

Surgical intervention through Strassman metroplasty provides an important decrease in the percentage of fetal loss (8-12%) compared to patients without surgical treatment (70-96%) [5]. Conventional transabdominal metroplasty seems to be a safe and efficient procedure in women with bicornuate uterine anomaly [5].

The actual benefit of metroplasty for a bicornuate uterus, however, has not been tested in a controlled clinical trial and metroplasty for now should be reserved for women in whom recurrent pregnancy loss occurs with no other identifiable cause [4].

In other centers, laparoscopic and hysteroscopy metroplasty has been shown to be a safe procedure and with all the additional benefits of minimally invasive surgery and it is a viable alternative to conventional open abdominal metroplasty [10]. Significant challenges exist in Sub-Saharan Africa in terms of access and skills in laparoscopic surgery which pose a significant drawback to the possibility of laparoscopic metroplasty and this case highlights this [10, 11].

All patients with Müllerian agenesis should be offered counseling and encouraged to connect with peer support groups where available. The psychological effect of the diagnosis of Müllerian agenesis cannot be underestimated. Many patients experience anxiety and depression, question their female identity, and grieve their infertility. These patients struggle with how to share their conditions with family members, peers, and romantic partners. Several cultural issues also come into play in the African setting with stigma directed to women who have had miscarriages and those with challenges getting children. This is an aspect of care that should be considered in cultural contexts similar to this case.

There is a paucity of data demonstrating reproductive success after metroplasty in cases of bicornuate uterus in Sub-Saharan Africa. In Kenya, not much work on management of Müllerian anomalies has been reported or published in recent years. This case report aims to increase the awareness of Müllerian duct abnormalities more specifically bicornuate uterus in cases of recurrent miscarriages, highlighting the diagnostic strategies to investigate and to demonstrate management options in low resource settings.

## Figures and Tables

**Table 1:** Antenatal profile.

**Profile**	**Value**
Hemoglobin	11.5 g/dl
Blood group	A+
Hepatitis BsAg	Negative
VDRL	Negative
HIV	Negative
